# Costs and longer-term savings of parenting programmes for the prevention of persistent conduct disorder: a modelling study

**DOI:** 10.1186/1471-2458-11-803

**Published:** 2011-10-14

**Authors:** Eva-Maria Bonin, Madeleine Stevens, Jennifer Beecham, Sarah Byford, Michael Parsonage

**Affiliations:** 1 Personal Social Services Research Unit, London School of Economics and Political Science, Houghton Street, London WC2A 2AE, UK; 2 Personal Social Services Research Unit, Cornwallis Building, University of Kent, Canterbury CT2 7NF, UK; 3 King's College London, Institute of Psychiatry, De Crespigny Park, London SE5 8AF, UK; 4 Centre for Mental Health, Maya House, 134-138 Borough High Street, London SE1 1LB, UK

## Abstract

**Background:**

Conduct disorders are the most common psychiatric disorders in children and may persist into adulthood in about 50% of cases. The costs to society are high and impact many public sector agencies. Parenting programmes have been shown to positively affect child behaviour, but little is known about their potential long-term cost-effectiveness. We therefore estimate the costs of and longer-term savings from evidence-based parenting programmes for the prevention of persistent conduct disorder.

**Methods:**

A decision-analytic Markov model compares two scenarios: 1) a 5-year old with clinical conduct disorder receives an evidence-based parenting programme; 2) the same 5-year old does not receive the programme. Cost-savings analysis is performed by comparing the probability that conduct disorder persists over time in each scenario, adopting both a public sector and a societal perspective. If the intervention is successful in reducing persistent conduct disorder, cost savings may arise from reduced use of health services, education support, social care, voluntary agencies and from crimes averted.

**Results:**

Results strongly suggest that parenting programmes reduce the chance that conduct disorder persists into adulthood and are cost-saving to the public sector within 5-8 years under base case conditions. Total savings to society over 25 years are estimated at £16,435 per family, which compares with an intervention cost in the range of £952-£2,078 (2008/09 prices).

**Conclusions:**

Effective implementation of evidence-based parenting programmes is likely to yield cost savings to the public sector and society. More research is needed to address evidence gaps regarding the current level of provision, longer-term effectiveness and questions of implementation, engagement and equity.

## Background

Conduct disorders, defined as "a repetitive and persistent pattern of dissocial, aggressive, or defiant conduct" [1], are the most common childhood psychiatric disorders with a prevalence of 4.9% for children aged 5-10 in Great Britain [2]; about three times as many suffer from non-clinical conduct problems [3]. Based on Office for National Statistics mid-2009 population estimates, over 29,000 children in England aged 5 have severe conduct problems.

The costs to society are high. Childhood behaviour problems are linked to later delinquency and criminality and lead to adulthood antisocial personality disorder in about 50% of cases [4]. The annual cost of conduct disorder-related crime in England may be as high as £22.5bn, while the lifetime cost for a single prolific offender may be as high as £1.1-1.9m [3]. In a follow-up study of London school children [5], costs associated with severe childhood conduct disorder were distributed across many public sector agencies, such as the National Health Service and the Department for Education, and by age 28 were 10 times higher than for children with no conduct problems. Potential savings from early intervention have been estimated at £150,000 per child [6].

Parenting behaviours may mediate environmental and other risk factors of conduct disorder [3]. The most successful parenting programmes targeted at parents of children with or at risk of developing conduct disorder are designed to improve parenting styles and parent-child relationships, in turn having positive effects on child behaviour [7]. A recent meta-analysis of randomised controlled trials found significant differences in parent and independent outcome reports of child behaviour, showing favourable results for the parenting programmes [7]. Parenting programmes have also been shown to reduce symptoms of ADHD, improve educational attainment, prevent non-intentional child injury, and improve mothers' mental health [8].

Our analysis does not aim to model any specific programme, but rather a 'generic' parenting intervention, drawing on data on a variety of evidence-based programmes that are likely to be implemented in the English context. While there are many different parenting programmes, administered in a variety of formats, often they are group-based lasting between 1.5 and 2.0 hours per week over 8-12 weeks [8]. A review informing the National Institute for Health and Clinical Excellence (NICE) guidance on parenting programmes suggests that these can be roughly divided into those focussing on the parent-child relationship and behavioural approaches, with the latter more likely to have been tested in clinical trials [9]. Examples are the Positive Parenting Program (Triple P, [10]) and Incredible Years (for example Edwards and colleagues, [11]).

Providing interventions is necessarily associated with costs. If the intervention proves effective, however, it may result in reduced support needs which can save money in the short as well as the longer term. A recent review of the economic evidence on parenting programmes [12] found three UK studies looking at short-term costs and effectiveness. A small pilot which targeted children aged 2-10, compared an intensive psychological intervention with treatment as usual in Child and Adolescent Mental Health Services and found no significant differences in costs and effects [13]. Similarly, Harrington and colleagues [14] found no significant differences between group-based parenting programmes for children aged 3-5 in a hospital and community setting, and the authors conclude that the location of treatment is less important than the services offered. Edwards and colleagues [11] found the Incredible Years (IY) programme to be cost-effective in the short-term with an incremental cost-effectiveness ratio of £73 per point improvement on the Eyberg Child Behaviour Inventory intensity score, and that service use reduced over time along with sustained effects on child behaviour measured 18 months later [15].

There is, however, a lack of evidence on the longer-term costs and benefits of parenting programmes from controlled trials. The evaluation of the multifaceted Perry Preschool Program suggests a possible long-term impact on conduct disorder, criminal behaviour and employment in a US context, with estimated social rates of return of up to 10% by age 40 [16]. A recent study from Ireland found that the IY parenting programme may offer a rate of return on investment to society of between 4.6% and 13.3%, examining savings from reduced imprisonment, use of special education services and duration of unemployment [17].

To estimate the potential costs of and longer-term savings from parenting programmes in England we present the results of a decision-analytic model from a public sector and a societal perspective. We discuss strengths and limitations of the model and highlight the need for future research to strengthen the evidence base.

## Methods

### Study design

**Figure 1:**
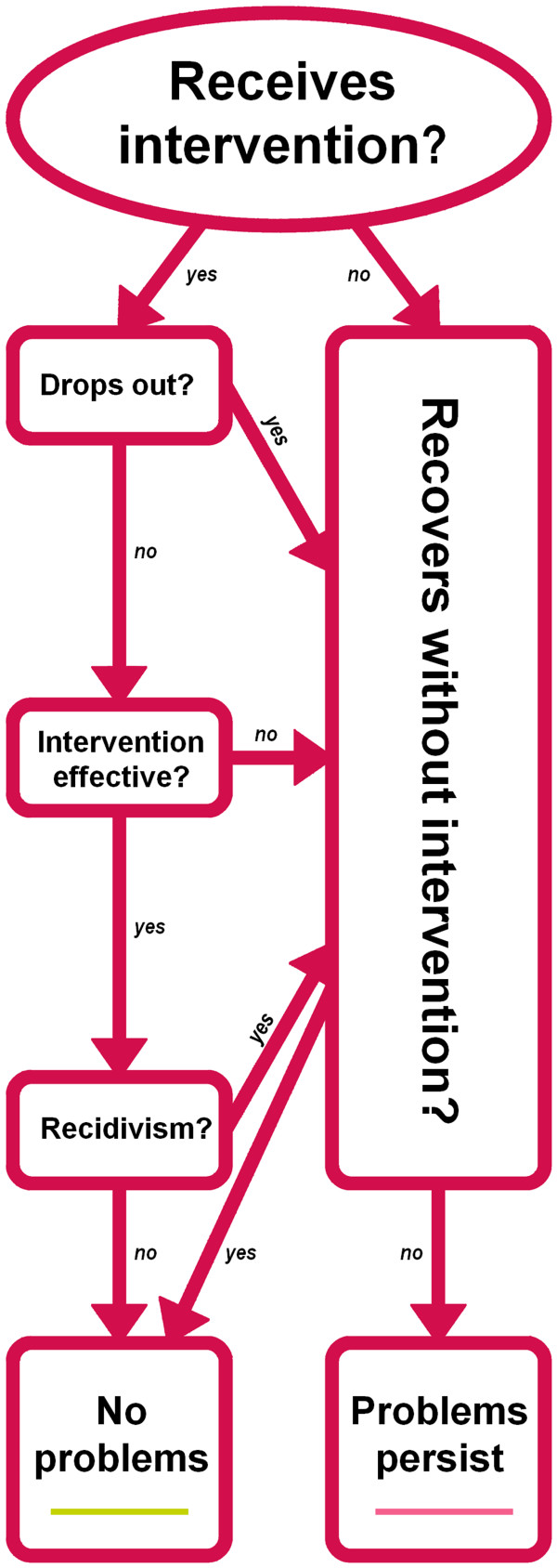
**Model of prevention of persistent conduct disorder**.

**Figure 2:**
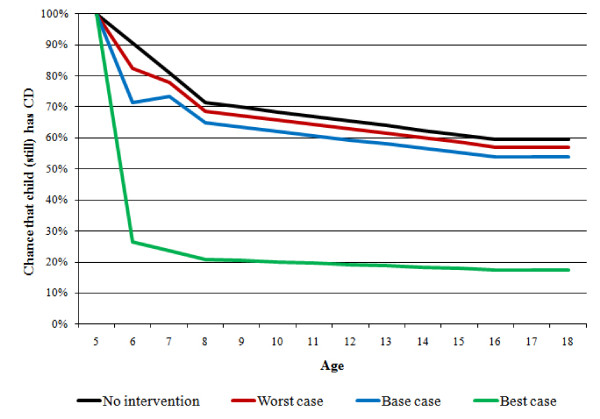
**Chance of conduct disorder persisting to later ages in a 5-year old with conduct disorder (4 scenarios)**.

Our decision-analytic model compares the costs incurred in two scenarios: 1) a 5-year old with clinical conduct disorder receives an evidence-based parenting programme and 2) the same 5-year old does not receive an intervention. Figure 1 shows a simplified model, summarising this process over time. The model covers 25 years ('Markov periods') and in each Markov period, there is a chance that conduct disorder will resolve without (further) intervention, thus following the 'natural course of conduct disorder' described below (see 'no intervention' case in Figure 2).

Costs are assigned based on the probability of the child's conduct disorder persisting over time. If the intervention makes it less likely that conduct disorder persists as the child ages, the model will show cost savings compared to the no-intervention scenario. Analyses were performed using TreeAge Pro 2007 and Excel 2003.

### Model parameters

Extensive literature searches were performed using data bases such as the National Health Services Economic Evaluation Database, the Cochrane Database of Systematic Reviews, PsycINFO, Medline and Google Scholar. In addition, Google searches were used to find unpublished papers and grey literature. Using systematic reviews wherever possible, we abstracted the data used to populate the model. Parameters used for the base case and sensitivity analysis are presented in Table 1.

**Table 1:** Model parameters and sensitivity analysis

***Parameter***	***Base case***	***Best case***	***Worst case***
**Drop-out**	44%	6%	60%
**Effectiveness**	34%	68%	20%
**Recidivism**	50%	0%	50%

#### Natural course of clinical conduct disorder

The model focuses on the impact of parenting interventions on childhood conduct disorder that persists into adulthood. In a study of UK children, Richman and colleagues [18] reported that about 60% of those showing behavioural problems at age 3 still exhibit these problems at age 8 and a review of the evidence [19] suggests that approximately 50% will continue to show problematic behaviours in adulthood. Assuming straight lines between these points and basing the analysis on only those children whose behaviour has not improved by the time they reach age 5, we estimate that without intervention, the chance that in a 5-year old with conduct disorder problems will persist beyond age 16 is about 59% (see 'no intervention' case in Figure 2).

#### Drop-out

The rates for parents not completing programmes vary greatly between types of interventions and indeed individual trials. While systematic reviews have reported drop-out rates of between 6% and 44% [20], they can be as high as 60% [21]. These values were used for the base (44%), worst (60%) and best (6%) cases. In the model, we assume that those who drop out will not experience a positive intervention effect and will not be replaced by other parents.

#### Effectiveness of parenting programmes

In this model, we do not focus on a specific programme but rather a range of programmes likely to be implemented in England. Our measure of the effectiveness of parenting programmes is a reduction in the number of clinical cases of conduct disorder. This was estimated using data from studies included in a recent systematic review of randomised controlled trials [7].

One commonly used measure of child conduct problems is the Eyberg Child Behaviour Inventory (ECBI, [22]). The ECBI problem score measures the number of difficult behaviours. The intensity score (ECBI-I), the outcome measure used for the model, measures how many times these behaviours occur, with higher scores indicating greater severity of behaviour problems.

Our analysis included studies where the mean ECBI intensity score (ECBI-I) for the control group was above the clinical cut-off point of 126 post-treatment and where the total number of study participants was at least 20. Based on post-treatment mean ECBI-I scores and standard deviations, we simulated the likely distribution of ECBI-I scores and estimated the proportion of children with clinically relevant conduct disorder post-treatment in the intervention and control groups. For each trial, the difference in the proportion of children in the clinical range post-treatment in the intervention and control groups is the estimated effect of the intervention for our model. On average, the parenting programmes resulted in a 34% reduction in clinical cases of conduct disorder from pre- to post-intervention (range 20% to 68%), over and above the reduction found for the control groups [D'Amico, F and Bonin, E 2010, unpublished data].

For each child receiving the intervention, the average reduction in the probability that conduct disorder persists to the next period (that is, from age 5 to age 6) is therefore 34%, but only if the family does not drop out - our base case scenario. The lowest reduction in conduct disorder cases (20%) was used as our effectiveness measure in the worst-case scenario, and the highest (68%) in the best-case scenario (see Table 1).

#### Recidivism

There is evidence from controlled trials of a sustained positive effect of parenting interventions on conduct disorder up to one year post-intervention, but studies reporting longer-term effects lack control groups [8,23]. Given the paucity of evidence, we assume that in the base case, 50% of children who initially improve to non-clinical levels of behaviour problems due to the intervention revert to their original level of behaviour problems after the first year post-intervention, by age 7. We assume no recidivism (0%) in the best case and that all children will revert to pre-intervention behaviour levels (recidivism rate of 100%) in the worst case scenario (see Table 1).

### Sensitivity analysis

Table 1 reports the parameters used to obtain results from the model for base, best and worst cases. The 'best case' shows the maximum potential cost savings from improved outcomes generated by the intervention, given our assumptions about the lowest rates of drop-out and recidivism and highest rate of intervention effectiveness. The 'worst case' analysis tests whether there are cost savings even using the least optimistic assumptions, that is, the highest rates of drop-out and recidivism and lowest rate of intervention effectiveness. Further sensitivity analysis included varying the mix of group and individual interventions provided in each case.

### Cost estimates

Average costs over and above those incurred by a child without conduct problems are presented at 2008/09 prices (see Table 2). Annual cost savings resulting from intervention have been calculated for each case (base, best, and worst) based on the probability that the child still has clinically significant conduct problems compared to the no-intervention scenario. Costs have been discounted at 3.5% throughout.

**Table 2:** Average annual cost of services per person with persistent conduct disorder, 2008/09 prices

***Budget***	***Age 5-10***	***Age 11-16***	***Age 17+***
**National Health Service**	£1,113^1^	£101^2^	£101^2^
**Social Services Department**	£157^1^	£63^3^	£63^3^
**Department for Education**	£882^1^	£1,202^2^	£0
**Voluntary sector**	£23^1^	£23^1^	£23^1^

^1 ^From Edwards and colleagues [11] and Harrington and colleagues [14] (uprated to 2008/09 prices), see Additional File 1: Appendix 1

^2 ^Scott and colleagues [5] (annualized, uprated to 2008/09 prices)

^3 ^Romeo and colleagues [26] (uprated to 2008/09 prices)

#### Parenting programmes

The costs of parenting programmes were estimated from details of five evidence-based and commonly used programmes submitted by programme developers to the NAPP Commissioning Toolkit [24] and include staff costs, overheads (capital, managerial and administrative), materials and additional items such as catering and childcare as well as the costs of training and supervision [25]. Given that we have information on only five programmes, the median cost was used to estimate the typical intervention cost for our model.

The cost of delivering group-based parenting programmes range from £282-£1,486 with a median of £952 per participant, while for individual interventions the costs range from £769-£5,642 with a median of £2,078. According to expert opinion submitted to NICE, about 80% of parenting programmes can be delivered in a group format [8] and this figure is used to weight the median costs. The expected intervention cost based on 80% group and 20% individual provision used for the model is therefore £1,177 per participant, and we vary the mix of provision (group vs. individual) in sensitivity analysis for each case.

#### Services

Table 2 shows the mean annual costs of conduct disorder to health and other services. In the model, these costs are incurred if a child has conduct disorder, and are thus saved if a parenting programme reduces the chance that a child has conduct disorder. Estimates for younger children (age 5-10) are based on combined data from two studies [11,14], weighted by the number of children in each study (see Additional File 1: Appendix 1). Scott and colleagues [5] show the costs to health services and education support per person with severe conduct disorder from age 10 to 28. These were used to estimate support costs from age 11-30, supplemented by primary care and social care costs presented by Romeo and colleagues [26].

#### Crime

Findings from the Christchurch Health and Development Study show that the 5% of people with the most severe childhood conduct disorder are responsible for 21.7% of all crimes [3]. Data on the number and type of offences committed by young people in England aged 10 to 25 were obtained from the 2006 cross-sectional sample in the Offending, Crime and Justice Survey [27]. These data were combined with Home Office unit costs [28,29], which include costs in anticipation of crime, as a consequence of crime and in response to crime. These data were used to calculate an average cost of crime per person for each year of age, given the presence of childhood conduct disorder (see Additional File 2: Appendix 2).

## Results

Figure 2 shows the probability that a child with conduct disorder at age 5 continues to show conduct problems at later ages. In the 'no intervention' scenario - based on the natural course of conduct disorder presented above - the chance that conduct disorder persists beyond age 16 is approximately 59%. The three intervention scenarios (base, best and worst cases) take into account that even when offered the intervention when the child is 5 years old, families may drop out of the programme. For those completing the intervention, the probability of conduct disorder persisting is reduced in line with our estimate of intervention effectiveness by age 6, but this effect is not sustained for a proportion of completers by age 7 (recidivism). In the model, all children who continue to have conduct disorder are assumed to follow the 'natural course of conduct disorder' path, which includes the chance that conduct disorder resolves without (further) intervention. Thus, the probability that conduct disorder persists beyond age 16 reduces to 54% in the base case scenario, 17% in the best case scenario and to 57% in the worst case scenario.

Table 3 shows potential total cost savings by agency or sector from providing a parenting programme to one family under base case assumptions. Total savings to the public sector amount to £5,837, while savings to wider society are £10,598. Towards the end of the table the net present value of savings to the public sector and from a societal perspective is shown. From a societal perspective, the intervention generates net savings (i.e. taking into account the cost of the intervention) of between £14,357 to £15,483 per family, depending on the mix of group and individual interventions, with almost half the savings accruing for reduced crime victim costs. The potential public sector savings over 25 years amount to between 2.8 and 6.1 times the intervention cost, the bulk of which are savings to the NHS and the criminal justice system.

**Table 3:** Present value of savings per family from a parenting programme over 25 years (base case)

*Budget*	*Present value*	*% of savings*
National Health Service	£2,195	13%
Social Services Department	£109	1%
Department for Education	£690	4%
Criminal Justice Service	£2,842	17%
***Public sector total***	***£5,837***	***36%***
		
Voluntary sector	£27	0%
Lost output (crime)	£2,197	13%
Victim costs (crime)*	£7,468	45%
Other crime costs**	£906	6%
***Other sectors/individuals total***	***£10,598***	***64%***
		
**Total savings**	**£16,435**	

**Intervention cost**		
- Group provision only	£952	
- 80% group, 20% individual provision	£1,177	
- Individual provision only	£2,078	
**Net present value of savings to public sector (to society)**		
- Group provision only	£4,885 (£15,483)	
- 80% group, 20% individual provision	£4,660 (£15,258)	
- Individual provision only	£3,759 (£14,357)	

* Intangibles

** Includes: costs in anticipation of crime, property damage and victim services

Table 4 reports the results of the scenario analysis in terms of net public sector savings per person in the first year following the intervention and total net public sector savings over 25 years, assuming 80% group provision and 20% individual provision. The base case scenario shows a positive return on the investment between five and eight years after the intervention. For the worst case scenario, where the proportion of children with persistent conduct disorder remains highest after the intervention, potential total savings are 42% lower compared to the base case. In the best case scenario, savings are 3.4 times as high as the base case in the first year following the intervention, and 7.6 times as high thereafter.

**Table 4:** Scenario analysis: Present value of net public sector savings per family from a parenting programme

	*Worst case*	*Base case*	*Best case*
Public sector savings year 1 (post-intervention)*	-£1,011	-£781	£152
Total public sector savings (25 years)*	£1,271	£4,660	£41,611

**Years to break even ***group provision only*	9	5	1
**Years to break even ***80% group, 20% individual*	9	6	1
**Years to break even ***individual provision only*	12	8	2

**Return to public sector ***multiple of intervention cost*	1.2 to 2.6	2.8 to 6.1	20.6 to 45.0
**Return to society ***multiple of intervention cost*	2.1 to 4.7	5.1 to 11.1	38.9 to 84.8

* Based on 80% group and 20% individual provision

## Discussion

Our study synthesizes the available evidence, in particular that from high-quality evaluations. We provide an up-to-date, comprehensive estimate of the potential savings from parenting programmes, using empirically derived estimates for the UK. Our analysis is based on the results of an extensive literature search, using estimates from systematic reviews where possible. Both the base case and sensitivity analyses rely on evidence-based, conservative assumptions. The cost of parenting programmes is based on data provided by developers rather than a hypothetical intervention.

Existing studies of service use and cost commonly include only a small number of children. By combining data from several studies we have tried to obtain a more robust estimate of costs for younger children, but as far as we are aware, only one study reports the longer term costs of conduct disorder in England [5], leading to uncertainty in the estimate of future savings.

While the cost analysis aimed to be comprehensive, a number of potential cost savings had to be excluded. Adults with a history of childhood conduct disorder experience a range of negative outcomes [30]. Overall, they are likely to earn lower-than-average wages [31] and have a higher probability of being unemployed, although those in employment may earn more than their peers [32]. Childhood conduct disorder is also associated with other adult mental health problems and disability [31,33]. However, there is insufficient evidence to suggest parenting programmes would have an impact on these outcomes. For the same reason, social security benefit receipt has also been excluded, although these costs may be substantial [16,26].

We have also had to exclude from the model other potential positive effects of the intervention such as impacts on the child's social network (parental mental health and employment effects, benefits to siblings and peers, intergenerational effects), those that overlap with other cost categories (educational attainment, teenage pregnancy, smoking, drug and alcohol abuse) and excess mortality due to higher rates of accidents and suicide [6,33]. While the aim was to look widely at all possible cost savings from providing parenting programmes for childhood conduct disorder, the lack of evidence means that the model is limited to reduced public expenditure and savings from the prevention of crime.

Problems arose from the absence of large longitudinal studies of children with conduct disorder in the UK. For example, to estimate crime rates, the cross-sectional Offending, Crime and Justice Survey has been interpreted as longitudinal data. Moreover, while the sample is weighted to be nationally representative, this survey excludes some potentially high offending groups such as those in prison, and offences such as homicide and sexual offences which will have high cost implications. We have also been unable to adjust for types of crime committed by those with conduct disorder; it may be that their pattern of offending behaviour is different from that of the rest of the population. Each of these factors will have an impact on the accuracy of our calculated savings to the criminal justice services but their inclusion is unlikely to change the overall findings.

Our model focuses on the savings to be achieved by reducing the probability of persistent conduct disorder for children with the most severe conduct problems. In analysis published elsewhere, we demonstrate that parenting interventions are cost-saving even when accounting for the fact that some children will improve but still exhibit behaviour problems [34]. The strongest evidence base both in terms of costs and intervention effectiveness, however, exists for children with clinical levels of conduct disorder and while conduct problems exist on a continuum and non-clinical levels may be associated with increased service costs compared to children without behaviour problems [5], the cost implications are by far largest for those with the most severe problems [3]. To illustrate this point, the 8% of males classed as 'prolific offenders' account for two thirds of all criminal convictions [3], and most of them will have exhibited behaviour problems since childhood. Furthermore, meta-analyses have found that studies of parenting programmes for children with clinical levels of behaviour problems have bigger effect sizes than those for children with sub-clinical levels [23,35], meaning that the biggest improvements can be achieved for this group. Consequently, reducing the prevalence of clinical conduct disorder will generate the largest savings and the largest benefit to wider society.

The model results are sensitive to changes in the assumptions about the natural course of conduct disorder. If more (fewer) people recovered without intervention, this would decrease (increase) the savings from the intervention.

Finally, although the intervention cost is derived from information provided by developers of evidence-based parenting programmes, there may be additional staff costs associated with engagement, preparation and follow-up support for families, and any additional organisational costs associated with rolling-out parenting programmes are not captured in the model.

## Conclusions

Investment in high quality, evidence based parenting programmes is likely to yield substantial cost savings for public services and broader benefits to society. However, as is the case with most interventions focussing on prevention, these savings will not be visible immediately; a long-term view is needed to ensure that these benefits can be achieved.

Further research is needed to complete the picture of the role parenting programmes can play in reducing the negative effects of conduct disorder. One important question is whether the positive effects of parenting programmes are sustained in the longer term. In the current literature, control groups are rarely included in follow-up assessments, often because they subsequently received the intervention.

Our results show that in the first year, parenting programmes may require a net investment, as savings are achieved over time. This highlights the importance of maintaining intervention effects and reducing drop-out rates, which might require additional resources. These are not trivial issues; for example, attrition may increase with length and demands of the programme, but attendance is positively related to greater effects [36].

Savings to the public sector accrue to several agencies but mainly to the NHS and the criminal justice system. However, parenting programmes are often provided by Local Authorities, through social workers or in school settings [37]. Funding decisions therefore need to take into account savings to all government budgets, and consideration given to the question of which agency is best placed to provide these programmes.

Since the publications of the 2006 NICE guidance on parenting interventions, the use of structured programmes has increased with 1,174 services delivering them in 2008/09 [38]. Most Local Authorities in England have mapped their provision of parenting programmes [37], however, little is known about their capacity, quality or impact and it is unlikely that all provision will be evidence-based. This major gap in our knowledge hampers attempts to ensure adequate service provision.

Finally, socially disadvantaged families may be exposed to multiple risk factors for conduct disorder, but at the same time they may be harder to engage in parenting programmes and although they may experience positive effects, these may be smaller than for less disadvantaged groups [36]. Future research is needed to address the questions of equity and targeting of interventions, and the implications for cost-effectiveness.

## Competing interests

JB and MS are partly funded by the National Academy for Parenting Research (funded by the Department of Education). This analysis was funded by the Department of Health and the Department for Education through the National Academy for Parenting Research based at the Institute of Psychiatry, King's College, London. All authors have completed the Unified Competing Interest form at http://www.icmje.org/coi_disclosure.pdf (available on request from the corresponding author) and declare: no support from any organisation for the submitted work except for funding as stated above; no financial relationships with any organisations that might have an interest in the submitted work in the previous three years, no other relationships or activities that could appear to have influenced the submitted work.

## Authors' contributions

EB developed the model, analysed and interpreted the results, wrote the manuscript, contributed to study design, literature search and appraisal and is guarantor. MS searched and appraised the literature and contributed to study design, analysis and interpretation of results and writing the manuscript. JB revised the manuscript, contributed to study design and advised on issues relating to the model and data analysis. SB contributed to study design and revising the manuscript and advised on issues relating to the model and data analysis. MP contributed to study design and revising the manuscript and advised on issues relating to the model and data analysis. All authors read and approved the final draft.

## Pre-publication history

The pre-publication history for this paper can be accessed here:

http://www.biomedcentral.com/1471-2458/11/803/prepub

## Supplementary Material

Additional file 1 **"Cost of conduct disorder to the public and voluntary sectors"**. Describes how the costs to public and voluntary sectors were derived from existing literature and operationalized in the model. Click here for file

Additional file 2 **"Cost of crime related to conduct disorder"**. Describes how the costs of crime used in the decision-analytic model were calculated from the Offending, Crime and Justice Survey and Home Office unit costs of crime. Click here for file
