# Supplementary material for: Costs and longer-term savings of parenting programmes for the prevention of persistent conduct disorder: a modelling study
Source: BMC Public Health. 2011 Oct 14;11:803. doi: 10.1186/1471-2458-11-803 (PMC3209459; doi:10.1186/1471-2458-11-803)
Supplement: Additional file 1 — "Cost of conduct disorder to the public and voluntary sectors". Describes how the costs to public and voluntary sectors were derived from existing literature and operationalized in the model. [file 1471-2458-11-803-S1.DOC]

# Appendix 1: Cost of conduct disorder to the public and voluntary sectors

The costs of services for children with conduct disorder, over and above the costs incurred by children without conduct problems, were calculated based on baseline data from two RCTs [1, 2]. Tables 1 and 2 show how the weighted average costs were obtained for each sample.

Table 1: Cost data from Edwards and colleagues (2003/04)

|  | ***Intervention group (n=73)*** | ***Control group (n=43)*** | ***Weighted average cost (n=116)*** |
| --- | --- | --- | --- |
| **Primary care** | £86 | £78 | £83 |
| **Hospital services** | £191 | £81 | £150 |
| **Special education** | £555 | £255 | £443 |
| **Social Services** | £58 | £60 | £59 |

Table 2: Cost data from Harrington and colleagues (1998/99)

|  | ***Community group (n=61)*** | ***Hospital group (n=57)*** | ***Weighted average cost (n=116)*** |
| --- | --- | --- | --- |
| **Psychiatric services** | £1,379 | £997 | £1,194 |
| **Other NHS costs** | £321 | £285 | £304 |
| **Social Services** | £120 | £290 | £202 |
| **Special education** | £1,513 | £761 | £1,150 |
| **Voluntary services** | £29 | £10 | £20 |

Table 3 shows how data from the two trials were combined and uprated to 2008/09 costs for the model. To obtain the marginal annual costs for children with conduct disorder, the ratio of costs for children with conduct disorder to the costs for children without conduct problems as reported by Scott and colleagues [3] was applied to the combined, weighted average cost from these trials.

Table 3: Costs categorised for the model

|  | ***Edwards*** | ***Harrington*** | ***Combined 2008/09*** | ***Ratio no CD : severe CD*** | ***Marginal annual*** |
| --- | --- | --- | --- | --- | --- |
| **National Health Service** | £233 | £1,498 | £1,255 | 0.11 | £1,113 |
| **Social Services Department** | £59 | £202 | £179 | 0.12 | £157 |
| **Department for Education** | £443 | £1,150 | £1,003 | 0.12 | £882 |
| **Voluntary sector** | n/a | £20 | £26 | 0.12 | £23 |

# References

1. Edwards RT, Ceilleachair A, Bywater T, Hughes DA, Hutchings J: **Parenting programme for parents of children at risk of developing conduct disorder: cost effectiveness analysis**. *BMJ* 2007, **334**(7595):682.

2. Harrington R: **Randomised comparison of the effectiveness and costs of community and hospital based mental health services for children with behavioural disorders**. *BMJ* 2000, **321**:1047-1050.

3. Scott S, Knapp M, Henderson J, Maughan B: **Financial cost of social exclusion: follow up study of antisocial children into adulthood**. *BMJ* 2001, **323**(191).
