# Supplementary material for: Costs and longer-term savings of parenting programmes for the prevention of persistent conduct disorder: a modelling study
Source: BMC Public Health. 2011 Oct 14;11:803. doi: 10.1186/1471-2458-11-803 (PMC3209459; doi:10.1186/1471-2458-11-803)
Supplement: Additional file 2 — "Cost of crime related to conduct disorder". Describes how the costs of crime used in the decision-analytic model were calculated from the Offending, Crime and Justice Survey and Home Office unit costs of crime. [file 1471-2458-11-803-S2.DOC]

# Appendix 2: Cost of crime related to conduct disorder

Data on offending was drawn from the 2006 cross-sectional sample from the Offending, Crime and Justice Survey [1]. This sample includes over 5,000 individuals aged 10-25 (51% female, 49% male) and is weighted to be nationally representative. It excludes people in institutions such as prisons and the homeless. It includes self-reported offences over the past 12 months, but does not cover all offences. Homicide and sexual offences are excluded.

According to previous research [2], the 5% with the most severe conduct problems commit 21.7% of crimes. Based on this information, we calculated the average marginal number of offences per person for young people with conduct disorder, i.e. the average number over and above offences committed by young people without conduct problems. The results are shown in table 1.

Table 1: Average number of offences related to conduct disorder per person

| ***Type of offence*** | ***Age*** | | | | | | | | | | | | | | | |
| --- | --- | --- | --- | --- | --- | --- | --- | --- | --- | --- | --- | --- | --- | --- | --- | --- |
|  | **10** | **11** | **12** | **13** | **14** | **15** | **16** | **17** | **18** | **19** | **20** | **21** | **22** | **23** | **24** | **25** |
| **Stole a vehicle** | 0.00 | 0.00 | 0.00 | 0.00 | 2.59 | 0.10 | 0.08 | 0.44 | 0.09 | 0.01 | 0.03 | 0.01 | 0.01 | 0.12 | 0.04 | 0.00 |
| **Tried to steal a vehicle** | 0.00 | 0.00 | 0.00 | 0.02 | 0.03 | 0.04 | 0.06 | 0.15 | 0.00 | 0.00 | 0.00 | 0.00 | 0.00 | 0.00 | 0.01 | 0.01 |
| **Stole off outside of vehicle** | 0.00 | 0.00 | 0.00 | 0.04 | 0.21 | 0.27 | 0.29 | 0.19 | 0.03 | 0.08 | 0.12 | 0.01 | 0.02 | 0.00 | 0.00 | 0.00 |
| **Stole from inside vehicle** | 0.00 | 0.00 | 0.12 | 0.00 | 0.33 | 0.30 | 0.04 | 0.11 | 0.03 | 0.01 | 0.02 | 0.02 | 0.00 | 0.00 | 0.00 | 0.00 |
| **Vehicle damage** | 0.00 | 0.08 | 0.02 | 0.05 | 0.28 | 0.46 | 0.31 | 0.08 | 0.09 | 0.20 | 0.12 | 0.05 | 0.02 | 0.69 | 0.07 | 0.00 |
| **Domestic burglary** | 0.00 | 0.00 | 0.02 | 0.01 | 0.05 | 0.02 | 0.03 | 0.00 | 0.03 | 0.00 | 0.01 | 0.00 | 0.01 | 0.01 | 0.00 | 0.00 |
| **Commercial burglary** | 0.00 | 0.00 | 0.00 | 0.04 | 0.49 | 0.42 | 0.14 | 0.04 | 0.00 | 0.10 | 0.04 | 0.00 | 0.17 | 0.00 | 0.00 | 0.00 |
| **Damaged Property** | 0.00 | 0.03 | 0.25 | 0.17 | 0.71 | 1.23 | 0.86 | 0.08 | 0.42 | 0.23 | 0.11 | 0.19 | 0.25 | 0.09 | 0.00 | 0.07 |
| **Commercial robbery** | 0.00 | 0.00 | 0.00 | 0.00 | 0.01 | 0.01 | 0.00 | 0.00 | 0.00 | 0.00 | 0.01 | 0.00 | 0.00 | 0.00 | 0.00 | 0.00 |
| **Personal robbery** | 0.00 | 0.00 | 0.00 | 0.00 | 0.01 | 0.00 | 0.00 | 0.00 | 0.00 | 0.00 | 0.00 | 0.00 | 0.00 | 0.00 | 0.00 | 0.00 |
| **Theft from person** | 0.10 | 0.00 | 0.03 | 0.03 | 0.04 | 0.01 | 0.04 | 0.00 | 0.00 | 0.00 | 0.04 | 0.00 | 0.05 | 0.00 | 0.00 | 0.00 |
| **Theft from work** | 0.00 | 0.00 | 0.00 | 0.00 | 0.04 | 1.03 | 0.27 | 1.74 | 0.76 | 0.66 | 1.22 | 0.57 | 0.66 | 1.00 | 1.02 | 0.50 |
| **Theft from school** | 0.25 | 0.06 | 0.44 | 0.70 | 1.34 | 2.08 | 1.62 | 1.39 | 0.35 | 0.26 | 0.03 | 0.05 | 0.09 | 0.01 | 0.14 | 0.01 |
| **Shoplifting** | 0.00 | 0.25 | 0.19 | 0.27 | 2.45 | 3.00 | 1.52 | 0.10 | 0.41 | 0.10 | 0.15 | 1.10 | 0.10 | 0.17 | 0.43 | 0.03 |
| **Other theft** | 0.25 | 0.00 | 0.22 | 0.07 | 0.58 | 0.69 | 0.32 | 0.20 | 0.13 | 0.08 | 0.13 | 0.00 | 0.07 | 0.01 | 0.22 | 0.00 |
| **Violence with injury** | 0.20 | 1.36 | 2.87 | 0.81 | 10.01 | 4.25 | 1.49 | 0.80 | 1.43 | 1.34 | 1.00 | 0.26 | 0.26 | 0.64 | 0.27 | 0.14 |
| **Assault, no injury** | 0.00 | 2.58 | 4.10 | 1.22 | 11.90 | 2.18 | 1.78 | 2.62 | 1.41 | 0.60 | 1.27 | 0.31 | 0.49 | 0.88 | 0.52 | 0.18 |

Unit costs were drawn from the 2000 and 2005 Home Office estimates [3, 4], uprated to 2008 / 09 prices using RPI. Table 2 below shows the type of offence as reported in the Offending, Crime and Justice Survey, and the Home Office unit cost applied. Victim costs are intangible costs, physical and emotional impact on direct victims of crime. Details on the method of unit cost estimation can be found in [3].

Table 2: Matching types of offences to unit costs

| **Type of offence** | **Unit cost applied** | **2008/09**  **Tangible costs** | **2008/09**  **Victim costs** |
| --- | --- | --- | --- |
| **Stole a vehicle** | Theft of vehicle | £3,935 | £943 |
| **Tried to steal a vehicle** | Attempted vehicle theft | £372 | £229 |
| **Stole off outside of vehicle** | Theft - not vehicle | £608 | £139 |
| **Stole from inside vehicle** | Theft from vehicle | £698 | £314 |
| **Vehicle damage** | Criminal damage | £464 | £556 |
| **Domestic burglary** | Burglary in a dwelling | £3,091 | £761 |
| **Commercial burglary** | Burglary not in a dwelling | £3,388 | £0 |
| **Damaged Property** | Criminal damage | £464 | £556 |
| **Commercial robbery** | Robbery or till snatch | £5,534 | £740 |
| **Personal robbery** | Robbery or mugging | £4,991 | £3,593 |
| **Theft from person** | Theft - not vehicle | £608 | £139 |
| **Theft from work** | Theft - not vehicle | £608 | £139 |
| **Theft from school** | Theft - not vehicle | £608 | £139 |
| **Shoplifting** | Theft from shop | £125 | £0 |
| **Other theft** | Theft - not vehicle | £608 | £139 |
| **Violence with injury** | Violence against the person | £5,817 | £6,450 |
| **Assault, no injury** | Common assault | £769 | £929 |

The resulting average marginal costs of crime per person with conduct disorder are shown in table 3.

Table 3: Costs of crimes related to conduct disorder (2008 / 09 prices)

| ***Age*** | ***Victim costs*** | ***Lost output*** | ***Health services*** | ***Criminal justice*** | ***Other**** | ***Total costs*** |
| --- | --- | --- | --- | --- | --- | --- |
| 10 | £1,168 | £332 | £270 | £565 | £128 | £2,465 |
| 11 | £9,515 | £2,931 | £2,145 | £3,311 | £93 | £17,995 |
| 12 | £19,196 | £5,842 | £4,371 | £6,858 | £383 | £36,650 |
| 13 | £5,613 | £1,671 | £1,243 | £2,191 | £371 | £11,089 |
| 14 | £67,131 | £19,878 | £14,957 | £24,047 | £10,454 | £136,467 |
| 15 | £26,456 | £7,647 | £5,994 | £10,549 | £3,085 | £53,732 |
| 16 | £10,508 | £2,960 | £2,224 | £4,399 | £1,629 | £21,720 |
| 17 | £7,337 | £2,058 | £1,397 | £3,412 | £2,379 | £16,583 |
| 18 | £9,445 | £2,756 | £2,105 | £3,627 | £787 | £18,720 |
| 19 | £8,148 | £2,381 | £1,879 | £3,167 | £620 | £16,194 |
| 20 | £6,787 | £1,995 | £1,499 | £2,797 | £666 | £13,744 |
| 21 | £1,844 | £510 | £384 | £817 | £315 | £3,871 |
| 22 | £2,056 | £573 | £411 | £1,029 | £682 | £4,751 |
| 23 | £4,794 | £1,309 | £973 | £1,903 | £831 | £9,810 |
| 24 | £2,095 | £585 | £422 | £1,087 | £483 | £4,672 |
| 25 | £980 | £274 | £205 | £470 | £134 | £2,063 |

* Includes costs in anticipation of crime, victim services and property-related costs (value of property stolen and value of property damaged or destroyed, net of value of property recovered)

# References

1. Home Office Research, Development and Statistics Directorate: **Social Research, Offending, Crime and Justice Survey 2006 [computer file]**. In *Offending Surveys and Research*. 2nd edition. Colchester: National Centre for Social Research and BMRB; 2008.

2. Sainsbury Centre for Mental Health: **The chance of a lifetime. Preventing early conduct problems and reducing crime**. London: Sainsbury Centre for Mental Health; 2009.

3. Brand S, Price R: **The Economic and Social Costs of Crime**. In *Home Office Research Study 217*. London: Home Office; 2000.

4. Dubourg R, Hamed J, Thorns J: **The economic and social costs of crime against individuals and households 2003/04**. In *Home Office Online Report 30/05*. London: Home Office; 2005.
